# Arts-based research in psychiatry: A way to the examination of the popular beliefs about mental disorders

**DOI:** 10.1192/j.eurpsy.2021.854

**Published:** 2021-08-13

**Authors:** F. Pavez

**Affiliations:** 1 Escuela Internacional De Doctorado, Universidad de Murcia, Murcia, Spain; 2 Suicide And Mental Health Research Group, University of Otago Wellington, Wellington, New Zealand

**Keywords:** medicine in the arts, social meanings of psychiatry, art-based research, depictions of mental disorders and psychiatry

## Abstract

**Introduction:**

Research about the depictions of psychiatry and mental disorders in popular culture has been scarce and often lacks systematized research strategies. However, this tendency has changed in the last few years and it is now possible to find articles which investigate the social representations of mental illness through the analysis of the media, music, films, and other artistic manifestations. One possible indication of the emerging relevance of this topic is the inclusion of the MeSH term ‘Medicine in the Arts’ in the database of the U.S. National Library of Medicine in 2018.

**Objectives:**

To understand prevalent ideas regarding mental illness and psychiatry in a specific time and place by using artistic and cultural productions as data sources.

**Methods:**

Content and Thematic Analysis

**Results:**

In this communication I present examples from the content and thematic analysis of 7,777 Spanish Punk Songs (1981-2010) referring to psychoses, suicide and related behaviors, and other interesting issues for the psychiatric field.

**Conclusions:**

The study of the products of popular culture can give us information about common ideas present in the social imaginary regarding mental disease. One advantage of this type of study is the public character of the data. In addition, the fact that artistic productions persist over time enables access to information which could not be gathered through other qualitative research designs. In this way, the products of popular culture could be seen as what I call ‘cultural fossils’, which can be ‘traced back’ to the historical time in which they were produced.

